# Biological pattern and transcriptomic exploration and phylogenetic analysis in the odd floral architecture tree: *Helwingia willd*

**DOI:** 10.1186/1756-0500-7-402

**Published:** 2014-06-27

**Authors:** Cheng Sun, Guoliang Yu, Manzhu Bao, Bo Zheng, Guogui Ning

**Affiliations:** 1 Key laboratory of Horticultural Plant Biology, Ministry of Education, College of Horticulture and Forestry Sciences, Huazhong Agricultural University, Wuhan 430070, P. R. China

**Keywords:** Biological pattern, *Helwingia Willd*, Phylogenetic analysis, Transcriptome

## Abstract

**Background:**

Odd traits in few of plant species usually implicate potential biology significances in plant evolutions. The genus Hel*wingia Willd*, a dioecious medical shrub in *Aquifoliales* order, has an odd floral architecture-epiphyllous inflorescence. The potential significances and possible evolutionary origin of this specie are not well understood due to poorly available data of biological and genetic studies. In addition, the advent of genomics-based technologies has widely revolutionized plant species with unknown genomic information.

**Results:**

Morphological and biological pattern were detailed via anatomical and pollination analyses. An RNA sequencing based transcriptomic analysis were undertaken and a high-resolution phylogenetic analysis was conducted based on single-copy genes in more than 80 species of seed plants, including *H. japonica*. It is verified that a potential fusion of rachis to the leaf midvein facilitates insect pollination. RNA sequencing yielded a total of 111450 unigenes; half of them had significant similarity with proteins in the public database, and 20281 unigenes were mapped to 119 pathways. Deduced from the phylogenetic analysis based on single-copy genes, the group of *Helwingia* is closer with Euasterids II and rather than Euasterids, congruent with previous reports using plastid sequences.

**Conclusions:**

The odd flower architecture make *H. Willd* adapt to insect pollination by hosting those insects larger than the flower in size via leave, which has little common character that other insect pollination plants hold. Further the present transcriptome greatly riches genomics information of *Helwingia* species and nucleus genes based phylogenetic analysis also greatly improve the resolution and robustness of phylogenetic reconstruction in *H. japonica*.

## Background

*Helwingia Willd* (*Helwingia japonica*), blooming from April to May and fruiting from August to October, is a dioecious shrub in Helwingiaceae. Currently, eight species were documented and five of them were discovered to distribute in China. It possesses high medical value, such as antibacterial, anti-inflammatory and blood lipid reducing effects [1]. The *Aquifoliales*, to which *H. Willd* belongs, have attracted researchers’ exceptional interest for their evolutionary histories. The floral architecture of *Helwingia* species significantly differ from all other plants. The inflorescences of both sexes are borne on the laminae of scale and foliage leaves [2]. However the potential biology significances and the evolutionary origin of this odd floral architecture are not well understood due to poorly available data of biological and genetic studies in *H. Willd*. The genomic sequences and trancriptomic information can hardly be found in public databases.

The advent of genomics-based technologies has revolutionized the past sequencing me thod, and transcriptome sequencing is an alternative way to rich the genome information. The newly developed high throughput sequencing technology is a powerful and cost-efficient tool for advanced research in many areas, including genome re-sequencing, micro-RNA expression profiling, and especially de novo transcriptome sequencing for non-model organisms [3-5]. Over the past years, Next-Generation Sequencing (NGS) has greatly accelerated our understanding of the complexity of gene expression, regulation and networks in both model and non-model organisms [6-9].

The current phylogenetic studies in plants are dominated by sequencing the plastid and/or nuclear ribosomal DNA [10]. However, the markers from plastid genome or ribosomal DNA have limitations in phylogenetic analysis at the high taxonomic levels [11]. Phylogenetic analysis based on Low-copy nuclear genes has a great potential to complement cpDNA/nrDNA based analysis, and greatly improves the resolution and robustness of phylogenetic reconstruction at all taxonomic levels [12]. Large-scale transcriptome sequencing has the potential utility in functional genes exploration, and it also provides rich information for fine phylogenetic analysis.

In the present study, the biological and morphological pattern is illustrated in detail and the transcriptome of *H. japonica* is firstly characterizated utilizing Illumina paired-end sequencing technology based on the Non-normalized cDNA of the leaves and flowers. A biological significance of odd floral architecture in *Helwingia* is suggested based on biological and morphological analysis. Combining the collected single-copy genes of more than 80 species, the phylogenetic analysis were undertaken and the preliminary conclusion of *H. Willd* ’s evolutionary location was evaluated via comparative analysis to two classified methods, the Cronquist [13] and the APG III [14,15] system, respectively. The conclusion robustly confirms that those single-copy genes can be exploited in the evolutionary and classification research.

## Results

### Morphological and biological pattern of *H. Willd*

**Figure 1:**
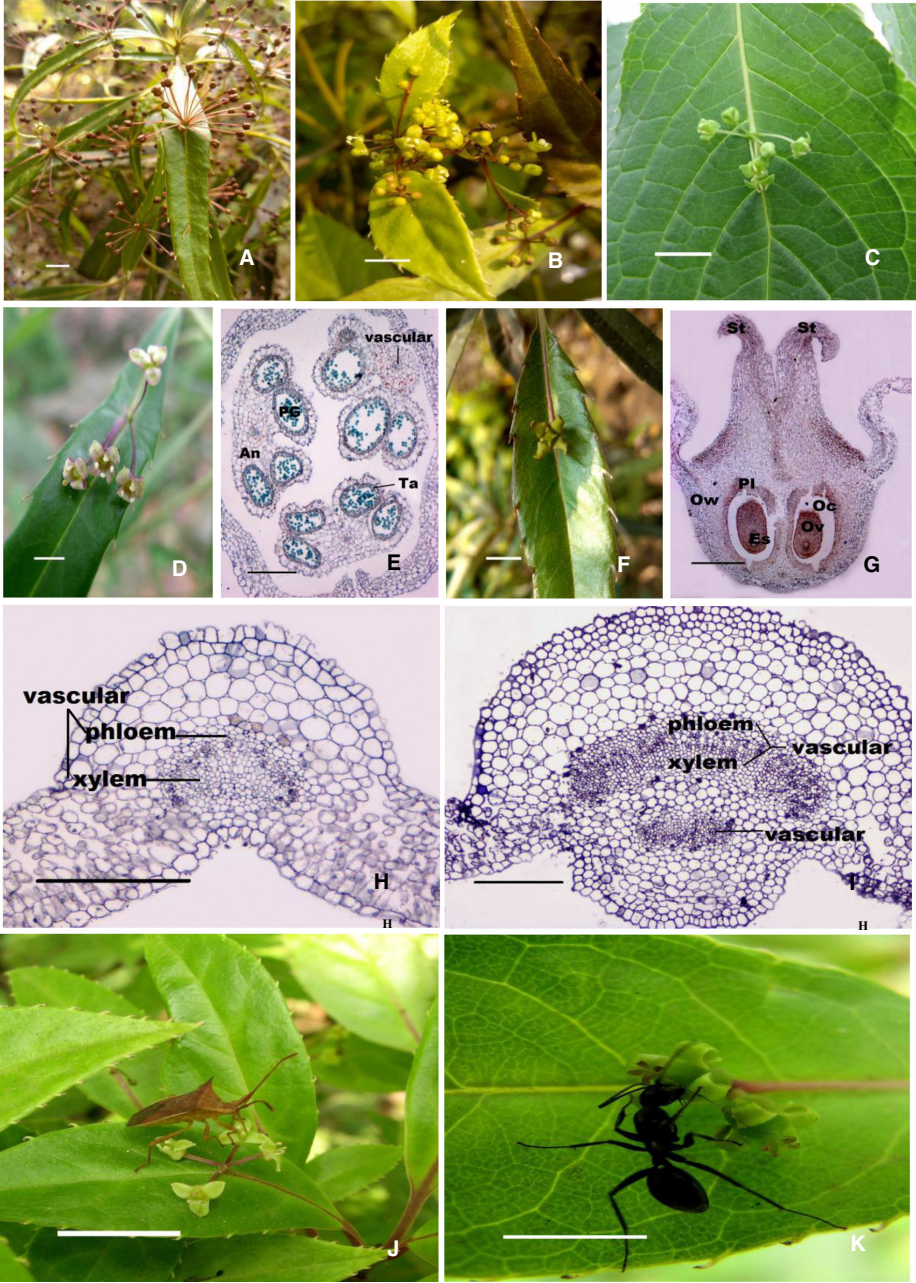
**Morphological and Biological pattern of H. Willd. (A-C)** Flower of H. chinensis, H. chinensis Batal. var. crenata and H. japonica respectively; (bar 1 cm). **(D-E)** Male Flowers of H. Chinensis and its’ transverse section, in which purple green flowers growing on midvein of leaf blade; (D: 5 mm; E: bar 0.1 mm). **(F-G)** Female Flower of H. Chinensis and its’ longitudinal section, in which the stigma was four divided; (F: bar 5 mm; G: bar 1 mm). **(H)** Transverse section of the top part of H. japonica leaf, in which one vascular is observed; (bar 1 mm) **(I).** Transverse section of the based part of H. japonica leaf, in which two vascular bundles are observed (obvious xylem, cambium and phloem are indicated);(bar 1 mm) **(J-K).** Stink Bug and Ant are visiting the Helwingia flowers in pollination.(bar 1 cm) Note: Po Pollen sac; An Anther; PG Pollen Granule; Ta Tapetum. OW Ovary Wall; Ov Ovule; Pl Placenta; OC Locule; ES Embryo Sac; St Stigma.

Five species of *H. Willd* growing in China show that all of them have epiphyllous inflorescences. During one growing season, the plant produces two type of leaves, the sterile foliage leaves (i.e., not inflorescence-bearing) and the fertile leaves (i.e., inflorescence-bearing). Of all the three presented species, the midrib color, until the inflorescence, of the fertile leaves is darker than that at the leaf tip. Simultaneously, the midrib between the petiole and the inflorescence is wider and thicker than the leaf tip (Figure 1A-C). The inflorescences of the fertile leaves are usually borne singly on the adaxial side of the midrib in the lower half of the lamina. They are imperfect flower, with only male or female reproductive organs. The flower have trimerous to pentamerous organs, possessing three to five petals, three to five calyx teeth and one flat floral disk. The male inflorescence of *H. chinensis* was a simple umbel composed of purple-green flowers; each of them has a small calyx with a two to ten-millimeter long pedicel, three to five petals and three to five stamens (Figure 1D). Like other common plants, transverse sections analysis of male flower show that it has butterfly-like anthers, and that its clinandrium have three layers, including the tapetum, mesoderm and epidermis, arranging from inside to outside, respectively (Figure 1E). The female inflorescence grows one to three flowers lacking of the stamens, they were nearly sessile or with short pedicels (less than 2 mm long), and their stigma have three to five divided carpels (Figure 1F). The ovary is inferior and unilocular with one pendant ovule. The stigma divides and the pollen tube grows along the guided tissue. The longitudinal section of its oval-shaped ovule indicated that the ovule had a thick integument and an oval-shaped nucellar (Figure 1G). The vascular anatomy of the mature leaf shows that a separated, adaxial vascular bundle departs from the leaf trace in the base of the petiole and leads to the inflorescence, in the mature fertile leaf. In this case, an abaxial collateral bundle curves around and opposes an adaxial collateral bundle (Figure 1I). The abaxial component continues as the midrib vascular bundle into the leaf tip (Figure 1H).

Continuous investigation during the whole flowering times verified that *H. Willd* was insect pollinated. Stinkbug (*Cletus punctiger*) and ant were the two most common types of insects visiting the flowers (Figure 1J-K). The leaves of *Helwingia* gave both the ant and stinkbug enough space to stand on when they were visiting the flowers. This phenomenon proved that the evolutionary odd floral architecture in Helwingiaceae made it greatly adapted to insect pollination.

### Comparison with the related species in traditional classification

The *Helwingia* genus was in Cornaceae (Table 1) according to the Cronquist traditional classification system [13]. The species in *Helwingia* genera had the similar biological and morphological pattern in flower characteristics, i.e. same number of the flower-merous and inferior ovary (Table 1). Such main characters also are observed in other genus species within Cornaceae. While it is not typical that there are many consistency characters among those species in the order level except for the ovary inferior trait (Table 1). Sub-class *Rosidae* Cornales include *Celastrales*, *Apliales*, *Rosales* and other orders, most species from them share the same number of flower-merous approximately. Compared with species in *Asteridae*, related to *Rosidae*, species in *Asteridae* tend to have superior ovary. There is no obvious similarity among those species at this level.

**Table 1:** Comparison of Helwingiacaea and related species under Cronquist system (1981)

***Class***	***Order***	***Family***	***Genus***	***Species***	***Characteristics***					
					***Flower unisexual***	***Calyx***	***Petal***	***Stamen***	***Ovary***	***Locules***
*Rosidae*	*Cornales*	*Cornaceae*	*Helwingia*	*Helwingia japonica*	*Yes*	*3-5*	*3-5*	*3-5*	*Inferior*	*3-5*
*Rosidae*	*Cornales*	*Cornaceae*	*Helwingia*	*Helwingia chinensis*	*Yes*	*3-5*	*3-5*	*3-5*	*Inferior*	*3-5*
*Rosidae*	*Cornales*	*Cornaceae*	*Helwingia*	*H. chinensis Batal.var.crenata*	*Yes*	*3-5*	*3-5*	*3-5*	*Inferior*	*3-5*
*Rosidae*	*Cornales*	*Cornaceae*	*Cornus*	*Cornus officinalis*	*No*	*4*	*4*	*4*	*Inferior*	*2*
*Rosidae*	*Cornales*	*Cornaceae*	*Swida*	*Swida alba*	*No*	*4*	*4*	*4*	*Inferior*	*2*
*Rosidae*	*Cornales*	*Cornaceae*	*Swida*	*Swida wilsoniana*	*No*	*4*	*4*	*4*	*Inferior*	*-*
*Rosidae*	*Cornales*	*Cornaceae*	*Aucuba*	*Aucuba chinensis*	*Yes*	*4*	*4*	*4*	*-*	*2*
*Rosidae*	*Cornales*	*Garryaceae*	*Dendrobenthamia*	*Dendrobenthamia japonica var. chinensis*	*No*	*-*	*-*	*-*	*Inferior*	*-*
*Rosidae*	*Cornales*	*Alangiaceae*	*Alangium*	*Alangium salviifolium*	*No*	*4-10*	*4-10*	*20-30*	*Inferior*	*1*
*Rosidae*	*Cornales*	*Davidiaceae*	*Davidia*	*Davidia involucrata*	*Yes & No*	*-*	*2-3*	*1-7*	*Inferior*	*6-10*
*Rosidae*	*Celastrales*	*Aquifoliaceae*	*Ilex*	*Ilex chinensis*	*Yes*	*4-5*	*4-5*	*4*	*Superior*	*4*
*Rosidae*	*Apliales*	*Araliaceae*	*Aralia*	*Aralia chinensis L.*	*No*	*5*	*5*	*5*	*-*	*5*
*Rosidae*	*Apliales*	*Araliaceae*	*Panax*	*Panax ginseng*	*No*	*5*	*5*	*5*	*-*	*2*
*Rosidae*	*Rosales*	*Grossulariaceae*	*Ribesa*	*Ribes nigrum*	*No*	*5*	*5*	*5*	*Inferior*	*-*
*Rosidae*	*Rosales*	*Grossulariaceae*	*Ribesa*	*Ribes davidii*	*Yes*	*5*	*5*	*5*	*-*	*2*
*Asteridae*	*Plantaginales*	*Plantaginaceae*	*Antirrhium*	*Antirrhium majus L.*	*No*	*-*	*1*	*4*	*-*	*-*
*Asteridae*	*Asterales*	*Asteraceae*	*Gerbera*	*Gerbera jamesonii*	*Yes & No*	*-*	*-*	*5*	*Inferior*	*1*
*Asteridae*	*Asterales*	*Asteraceae*	*Helianthus*	*Helianthus_annuum*	*Yes & No*	*2*	*Combined*	*5*	*Inferior*	*1*
*Asteridae*	*Solanales*	*Solanaceae*	*Solanum*	*Solanum tuberosum*	*No*	*5*	*5*	*5*	*Superior*	*-*
*Asteridae*	*Solanales*	*Solanaceae*	*Solanum*	*Solanum lycopersicum*	*No*	*5-7*	*5-7*	*5-7*	*Superior*	*2 to several*
*Dilleniidae*	*Ericales*	*Theaceae*	*Camellia*	*Camellia sinensis*	*No*	*5*	*5-6*	*Several*	*Superior*	*3*
*Dilleniidae*	*Ericales*	*Ericaceae*	*Rhododendron*	*Rhododendron ponticum*	*No*	*5*	*5*	*10*	*Superior*	*10*

### Paired-end sequencing and de novo assembly

**Figure 2:**
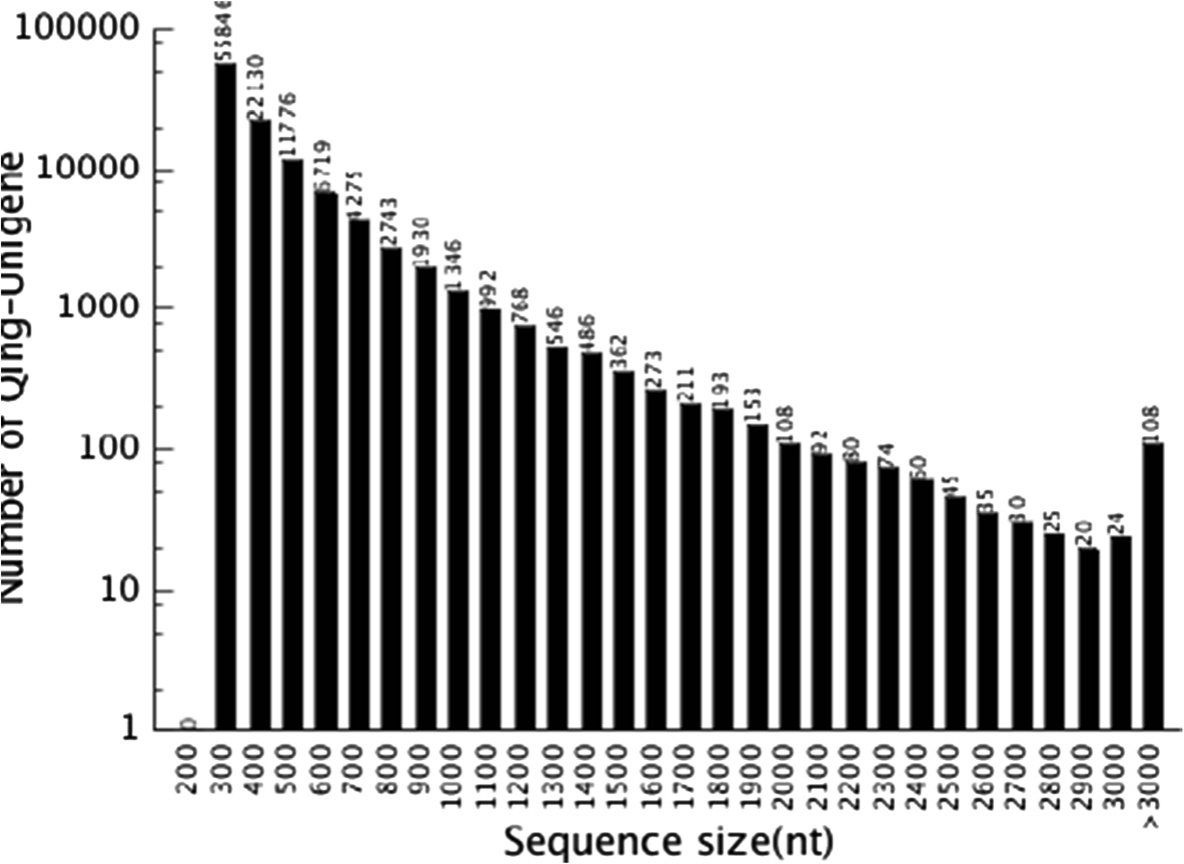
Assessment of assembly quality, which indicates distribution of unique mapped reads of the assembled unigenes.

A total of 40833338 raw reads with the length of 100 bp were generated from a 200 bp insert library using Illumina paired-end sequencing method. The raw reads were cleaned by removing adaptor sequences, empty reads, and low-quality sequences. On high-quality cleaned reads, a total of 928284 contigs, ranging from 50 to 4732 bp, were assembled with an average length of 127 bp and an N50 length of 99 bp. The contigs were then joined into scaffolds, based on paired-end information using “N” to represent unkown nucleotides between each two contigs. 228299 scaffolds were obtained with an average length of 265 bp (Table 2). The scaffold, ranging from 100 to 7239 bp, with an N50 length of 308 bp. Afterward, we used those paired-end reads again to fill the scaffold gaps to obtain unigenes with fewest Ns and could not be extended on either end. Finally the de novo assembly yields 111450 unigenes (Figure 2) with an average length of 400 bp and a total length of 44.6 Mb, in which the N50 length was 420 bp. The length of assembled unigenes ranged from 200 to 7246 bp. Among these unigenes, 21698 (19.47%) were greater than 500 bp long and 4685 (4.20%) were longer than 1 kb (Additional file 1). In addition, a total of 94406 (84.71%) unigenes showed no gap (data were not shown). The best-aligning results from unigenes quality evaluation and CDS prediction showed that 54853 out of 111450 unigenes (49.22%) had a BLAST homologous match against the public protein databases. For those unigenes had no hit in blast, the CDS were predicted by ESTScan. Finally, 59101 unigenes (53.03%) were oriented.

**Table 2:** **Overview *of the sequencing and assembly***

	***N50***	***Mean size***	***Total length***	***Total number***
***Read***	*—*	*90*	*3675000420*	*40833338*
***Contig***	*99*	*127*	*118117971*	*928284*
***Scaffold***	*308*	*265*	*60563756*	*228299*
***Unigene***	*420*	*400*	*44587120*	*111450*

### Function annotation and Gene Ontology classification

**Figure 3:**
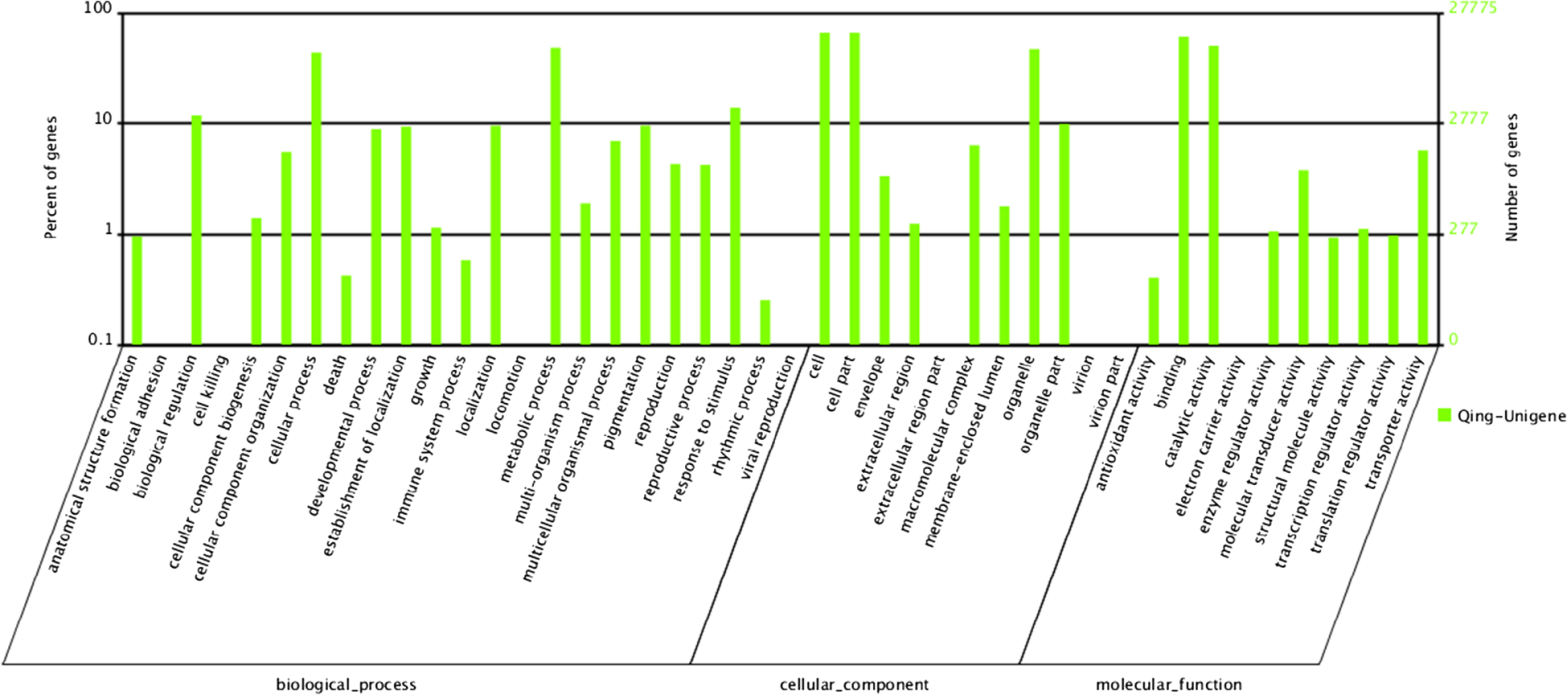
**Histogram presentation of GO classification of Helwingia japonica.** GO term assignment to the Helwingia transcripts in different categories of biological process, molecular function and cellular component. The right y axis indicates the number of genes in a category. The left y axis indicates the percentage of a specific category of genes in that main category.

Sequence similarity search indicated the retrieved proteins had the highest sequence similarities with given unigenes and a total of 54853 (49.22%) were annotated in this manner. To classify the function of the predicted *H. japonica* genes, Blast2GO successfully annotated 27775 sequences to 101010 GO terms out of the total 54853 sequences with BLAST matches. In many cases, multiple terms were assigned to the same unigene, this resulted in 50713 unigenes assigned to “biological process” category, 55542 to “cellular component” category and 34538 to “molecular function” category. Among the various biological process, “metabolic process” (13359, 26.34%) and “cellular process” (12121, 23.90%) were the most represented (Figure 3). The genes, involved in other important biological process such as response to stimulus and biological regulation, were also identified through GO annotations. Similarly, “cell” and “cell” part (both 18096, 32.58%) were prominently represented, whereas almost no genes were assigned to “virion” or “extracellular region”. Under the category of molecular function, “binding” (16719, 48.41%) and “catalytic activity” (13906, 40.26%) represented the majority of the category.

### Function classification by COG and KEGG

**Figure 4:**
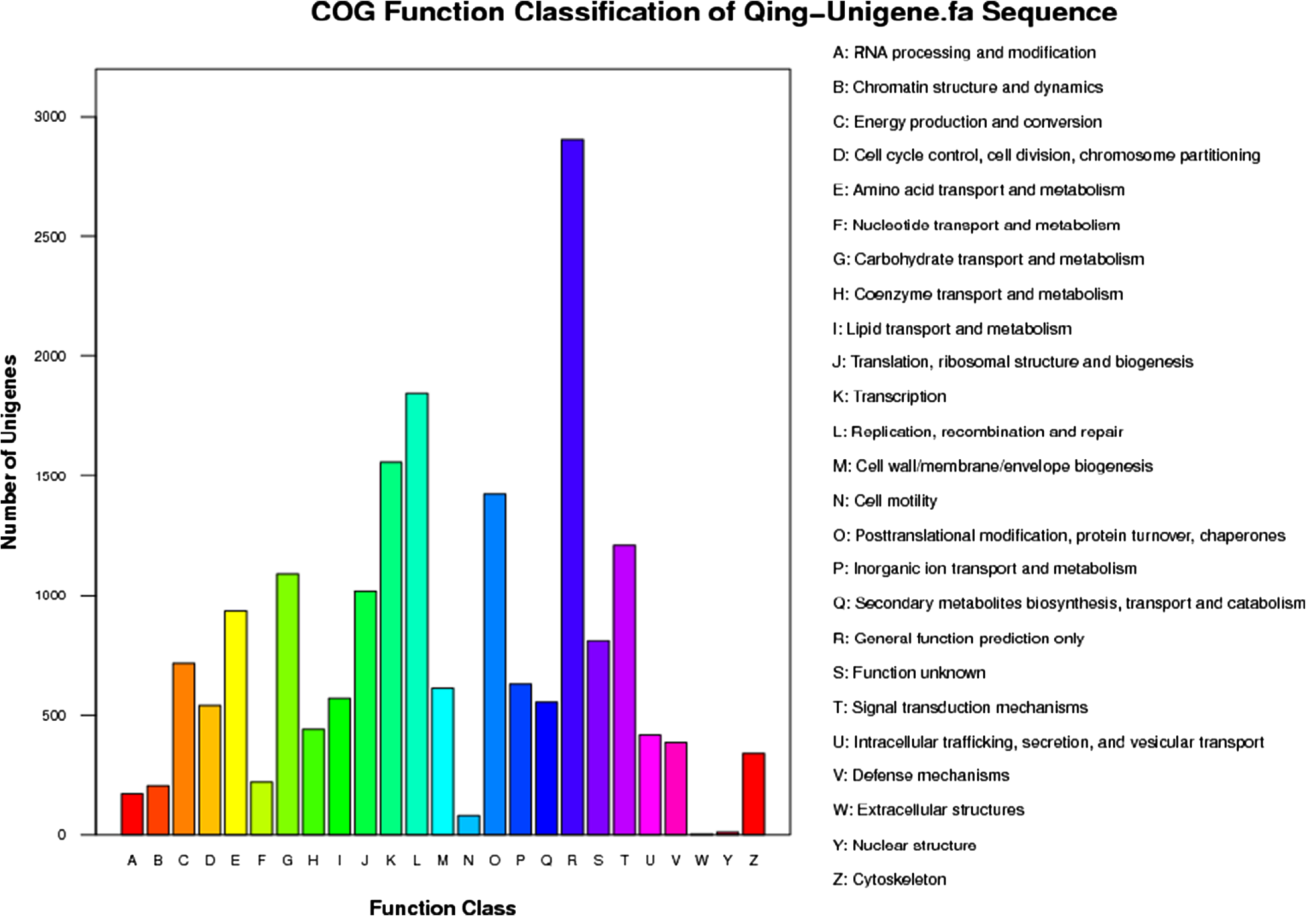
**Histogram presentation of COGs classification.** The histogram shows the distribution of sequences among different COG categories: out of 54853 blast hits, 27775 sequences have a COG classification among the 25 categories.

To further evaluate the completeness of annotation process, all unigenes were aligned to the COG database. 11732 sequences were assigned to the COG classifications (Figure 4). Among the 25 COG categories, the cluster for “general function prediction only” (2908, 15.58%) represented the largest group, followed by “replication recombination and repair” (1841, 9.87%), “transcription” (1558, 8.35%) and “posttranslational modification, protein turnover and chaperones” (1424, 7.63%), whereas only a few unigenes were assigned to “extracellular structures” and “nuclear structure”. 1211 unigenes were assigned to “signal transduction mechanisms”. Based on a comparison against the KEGG database using BlastX (e-values < 1.00E-05), out of the 111450 unigenes, 20281 unigenes (18.20%) can be mapped with 119 metabolic pathways (Additional file 2). 12481 unigenes have enzyme commission (EC) numbers, and were assigned to the metabolic pathways. The pathways with the most representations of unique sequences were the metabolic pathways (4610, 22.73%), followed by those related to plant-pathogen interaction (1657, 8.71%) and splicesome (1113, 5.49%).

### Shared single-copy genes in the solexa transcriptome assemblies and their Phylogenetic analysis

**Figure 5:**
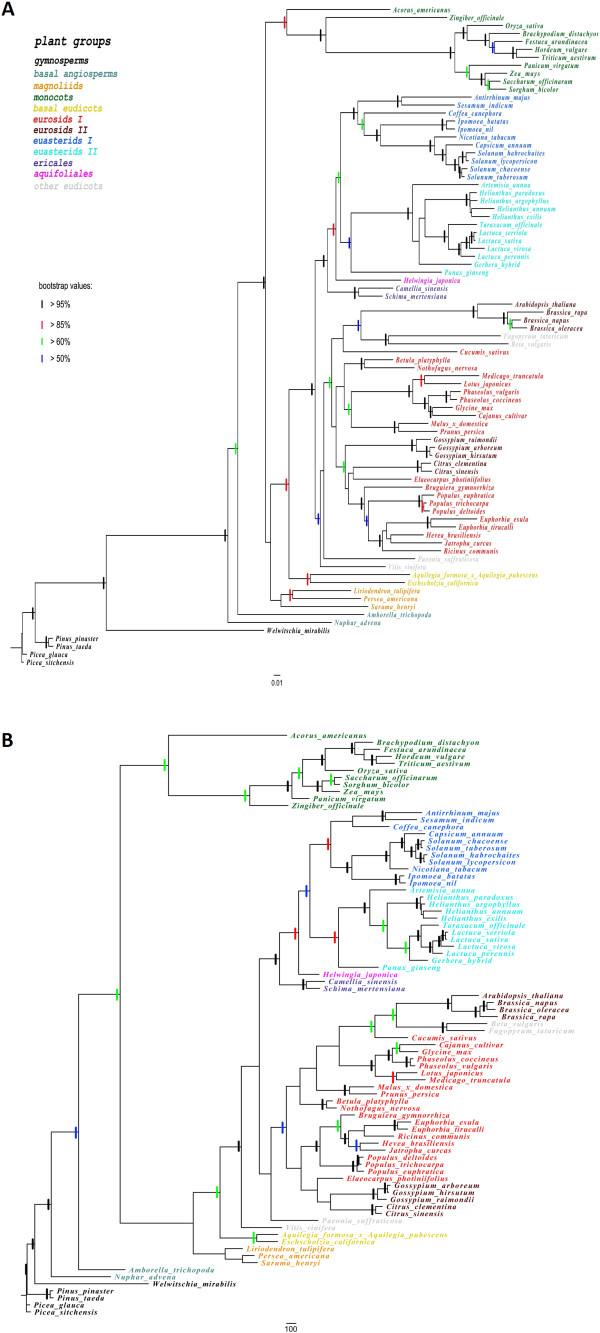
**Trees inferred from the maximum maximum likelihood analysis (A) and maximum parsimony analysis (B) of nucleotide sequences of 13 single-copy genes.** Picea sitchensis was used as the outgroup taxa for all analyses. Branch lengths are proportional to the number of expected nucleotide substitutions; scale bar corresponds to one substitution per hundred sites for the ML tree and to 100 changes for maximum parsimony tree. Non-parametric bootstrap values (greater than 50%) are indicated by the colored bars placed on branches.

13 confirmed shared sing-copy or low-copy nuclear genes [7,11] across the major lineages of angiosperm were used to screen their orthologs in 14 transcriptome assemblies, including the transcriptome assembly of *H. japonica* in this study (Additional file 3). Finally, two close related species, *H. japonica* and *Panax ginseng*, together with the other twelve species, were used to perform phylogenomic analysis. These fourteen species contain at least 6 single-copy homologous genes. For most of the thirteen single-copy genes, only one sequence with high similarity to a certain gene was found, which indicates that they are single-copy in the sampled species. As for the sampled transcriptome that had multiple significant blast hits to a certain gene, the orthologs were identified using the bidirectional blast method. Orthologs of most of these genes were found in all these transcriptomes. The collected sequences were added to the dataset reported before [7,11], and resulted in an 83-taxon nucleic acid sequence dataset containing 9159 characters (including gaps) (Additional file 4). The phylogeny was reconstructed using the maximum parisomny (MP) and maximum likelihood (ML) methods based on both nucleotide and amino acid sequences. The phylogenetic trees resulting from the two methods were similar to those inferred by the previously published studies based on those plastid sequences dataset [16-19] in topology and resolution though there were some differences in the placement of individual species between the MP and ML trees. Overall, the ML tree, with bootstrap values higher than 50 for most nodes in both nucleotide and amino acid based tree, shows improved resolution and increased bootstrap support compared to the MP tree (Figure 5).

### Classification of tested species using single or low copy nucleus genes and the similarity to APG III system

**Figure 6:**
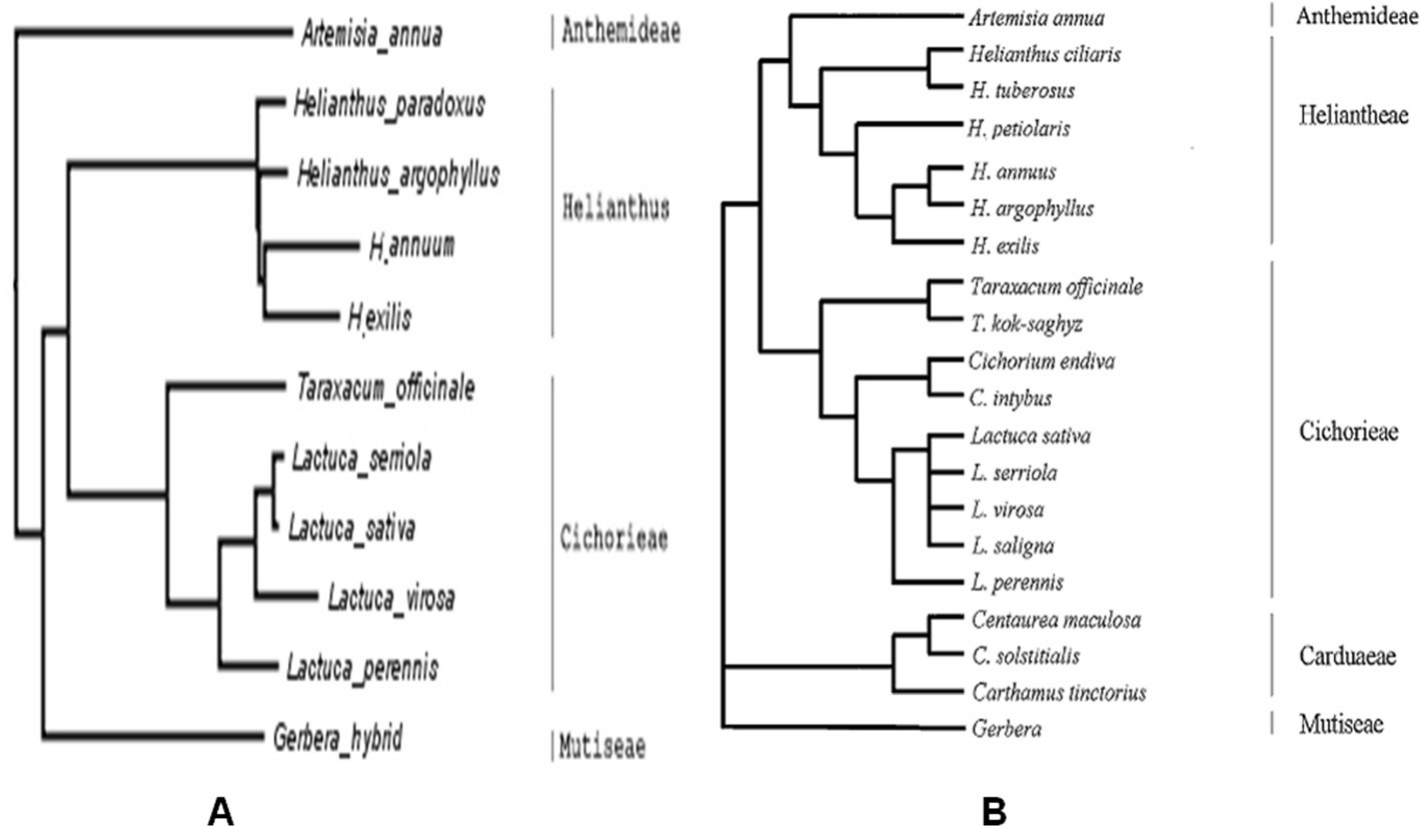
**Comparative analyses between phylogenetic relationships nuclear genes and plastid sequences. (A)** The interception of the phylogenetic relationships for the sampled lineages of Asteraceae from our ML tree based on 13-single copy nuclear genes. **(B)** Phylogenetic inferred relationships for the major lineages of Asteraceae based on ribosomal sequences from Rieseberg [20] Funk et al. [21], and Timme et al. [20-22].

The deduced phylogenetic trees are largely congruent between the MP and the ML methods. The resulting phylogeny topologies from ML tree shows improved resolution, which are greatly consistent to many previous phylogenetic studies based on plastid sequences at varying taxonomic levels (Figure 6). Our phylogenetic analysis based on the thirteen single-copy nuclear genes also supported that the group of *Helwingia* is closer with *Euasterids* II than *Euasterids* I (Figure 6). Our data also strongly suggests that the relationships between those clades of *Asterids*, including *Ericales*, *Lamiids* (ie. *Euasterids* I) and *Campanulids* (ie. *Euasterids* II), are sister relationships. The result of subgroups is also similarity to the APG III system that the *Helwingia* genus, belonging to *Aquifoliales,* is placed under the clade of *Campanulids*.

## Discussion

### Helwingia implicates a biological significance on Odd floral architecture

In *H. Willd,* the fusion of flower stalk to petiole might protect the flowers from rigid environments or shorten the distance of nutrition supplementation from leaves to flowers. In this study, the fusion pattern was carefully characterized by vascular anatomy. Floral adaptation to animal or abiotic pollination is considered to have been a key basis for the morphological diversification of angiosperms [23]. On common, for insect pollination, the animal size is usually much smaller than the flower size. *Helwingia* does not have big flowers, however their wide leaf blades provide the insects enough space to stand on when they visit the flowers, which further verifies how Helwingiaceae species present alternative pattern to adapt to insect pollination. In *H. Willd* the size of insects is much larger than that of flowers, so it is very important for Helwingiaceae to uphold the visiting insects with the leaves during pollination. Fossil evidence indicates that ants emerged in the Late Jurassic, 150 million years ago, while the flowering plants 100 million years ago [24,25]. Another *Helwingia* flower visiting insect – stinkbugs were assumed to emerge from the Late Triassic Epoch to the Middle Ages, but mostly in the Middle Ages [26]. Whether it is an evolution or an incident case to make the odd floral architecture, it gives us infinite cues.

### Transcriptome analysis riches genomics information of Helwingia species

Prior to this study, the limited numbers of sequences (only 35 ESTs) were deposited in GenBank. Our transcriptomic analysis yielded over 111 thousands of unigenes that could be matched to known genes via BLAST search, and many of them are involved in leaf and floral development. Those “non-blastable” sequences, to some extend, are inherent to the following categories: the non-coding cDNA fraction, lineage-specific genes and fast-evolving genes. This relative fraction of unigenes that did not have any BLAST hits may be an integral part of genomic data [7]. The transcriptome analysis of *H. japonica* might be more effective to get lots of genomic data and more helpful to deep the related researches in *Helwingia* species.

Comparison of assembled gene models and functional annotation (GO, COG, KEGG) indicates that we have sampled an expansive and diverse expressed gene catalog representing a large proportion of the genes expressed in *Helwingia.* The *Aquifoliale*s, which *H. Willd* belongs to, have attracted an great interest from evolutionary biologist. The presented transcriptomic data will be significantly important for further research on functional genomics.

### Transcriptomic data set effectively used to perform phylogenetic analysis

Plant phylogenetic studies has been dominated by using the plastid sequences (e.g. *matK*, *trnL-F* and *rbcL*) and/or nuclear ribosomal DNA (*18S*, *26S*, *ITS* and *ETS*) [10,27]. However, the markers from plastid genome or ribosomal DNA have limitations on phylogenetic utilities at high taxonomic levels [12]. Single or low-copy nuclear genes have a great potential, in compensation for cpDNA and nrDNA, to improve the resolution and robustness of phylogenetic reconstruction at all taxonomic levels. Transcriptomic data is a potential source of information for multigene phylogenetic analysis. Duarte et al. identified a set of single-copy genes shared between *Arabidopsis, Populus, Vitis* and *Oryza*[11]. Though many articles have reported about the evolutionary position of Helwingiaceae at varying taxonomic levels, none of them indicated the use of nuclear gene for phylogenetic analysis. Based on these single or low copy nuclear genes from more than 80 species, including Helwingiaceae, the phylogenetic tree inferred from the maximum parsimony and maximum likelihood analysis shown a higher resolution and node support. Our result is consistent with the privious hypothesis based on multiple plastid sequences.

### Phylogenetic analysis based on Helwingia nuclear genes is consistent with that based on plastid or ribosomal DNA sequences

Comparative analysis among varied classified system indicated that the tradition classification, the Cronquist system, was not quite consistent with our phylogenetic analysis. *Panax ginseng* was placed in *Rosidae* according to the traditional classification, whereas our phylogenetic analysis showed that *Panax ginseng* was indeed close to *H. japonica. H. Willd* was classified to Cornaceae in the systematics of Cornaceae, which is suggested remarkably unsettled and controversial by some researchers [28]. Many genera had been added to or removed from Cornaceae with time [29,30]. Currently, phylogenetic analyses based on plastid or ribosomal DNA datasets have consistently supported that Aquifoliaceae (Ilex only), Helwingiaceae (*Helwingia* only), and Phyllonomaceae (*Phyllonoma* only) were sisters within *Aquifoliales*[16,19]. The nuclear genes-based phylogenetic analysis here gives another solid evidence on it and confirm the phylogenetic location of *H. japonica*, which also presents the same trends as Duarte’s reports on some tested species using limited single or low-copy nuclear genes to perform phylogenetic analysis [11]. Additional, the result is also consistent with previou phylogenetic studies referred to Helwingiaceae based on plastid or ribosomal DNA [28,31]. Moreover, this presented subset produced well-resolved tree topologies similar to that inferred based on plastid or chloroplast sequences in many recent phylogenetic studies [7,17]. The phylogenetic tree presented in our study is somewhat similar with the circumscription of the genera of *Helwingia* in APG III (Angiosperm Phylogeny Group III) system. In APG III, the genera of *Helwingia* is placed within the order *Aquifoliales*, under the clade of *Campanulids*. In the MP and ML trees, *Helwingia* was both identified as more closely related to *Euasterids* II than to *Euasterids* I.

## Conclusions

The present study characterized the biological and transcriptomic patterns of *H. japonica*, a rare but vital species for research of evolutionary mechanism. The morphological and pollinated characteristics were elucidated and a comprehensive transcriptome is firstly developed. About 111450 assembled transcripts were obtained, with a half of them matching to known proteins. In addition, a phylogenetic analysis based on nuclear single or low-copy genes was proceeded, which has a great potential to complement cpDNA and nrDNA based phylogenetic analysis and enhances the resolution of plant phylogenetic reconstruction at all taxonomic levels. The *H. japonica* transcriptome and the collected low-copy nuclear genes dataset from more than 80 species reported in our study will rich the genomic resources for *Helwingia* and *Asterids* order. It will also be a significant contribution towards reconstruction of the plant phylogenetic tree.

## Methods

### Sample collection and preparation

Tissue samples of *H. Willd*, were collected from mature plants growing in both the Wuhan Botanical Garden (Hubei Province) and Mt. Lushan (Jiangxi Province) in China. Standard procedures of paraffin section analysis were according to our previous described method [32]. The sections were observed and photographed under appropriate magnifications using a NIKON research microscope.

### RNA isolation and cDNA library preparation for transcriptome analysis

Total RNA was isolated using TRIzol reagent (Invitrogen) according to the manufacturer’s instructions. mRNA was purified using oligo (dT) magnetic beads, and then fragmented into small pieces using divalent cations under elevated temperature. The cleaved RNA fragments were transcribed into first-strand cDNA using reverse transcriptase and random hexamer-primers, followed by second-strand cDNA synthesis using DNA polymerase I and RNaseH. After end repairing, adapter ligations were conducted. The products were purified and enriched with PCR to create the final cDNA library. All the experiments were undertaken in the Beijing Genome Institute (BGI, Shenzhen, China).

### Sequencing and de novo assembly

cDNA library was sequenced on an Illumina HiSeq2000 sequencing platform. The average size of inserts in the library was 200 bp, and it generates 100 bp PE reads [4]. Image deconvolution and quality value calculations were performed using Illumina HCS 1.1 software. De novo assembly was carried out using SOAPdenovo (http://soap.genomics.org.cn/soapdenovo.html) with the default settings [33] except with 29 K-mers after varied K-mers were firstly tested. Contigs without ambiguous bases were obtained by conjugating the K-mers in an unambiguous path. Next, SOAPdenovo connected the contigs using N to represent unknown sequences via the paired-end information to generate Scaffolds. Paired-end reads were used again for gap filling of scaffolds to get sequences with least Ns and cannot be extended on either end, which were finally defined as Unigenes. To obtain distinct gene sequences, the unigenes were clustered using the TGICL (TIGR Gene Indices Clustering) tools.

### Function annotations of transcriptome

Unigenes were aligned with the NCBI Nr and Swiss-Prot protein databases using BLASTx [34] with an E-value cut-off of 10^-5^ to evaluate the quality and predicted CDS. Unigenes that did not have significant hits in these databases were scanned using ESTScan [35]. Blast2GO [36] was used to obtain GO (Gene ontology) terms according to molecular function, biological process and cellular component (http://www.geneontology.org) based on BLASTx hits against the NCBI Nr database. Annotation with the COG and KEGG [37] pathways were also performed using BLASTx against the COG database and the Kyoto Encyclopedia of Genes and Genomes database.

### Phylogenetic analysis

For the phylogenetic analysis, a dataset of 13 single-copy genes from 69 taxa was collected from Duarte et al. [11]. The orthologs of these genes were screened by the BLAST search of corresponding *Arabidopsis* and *Oryza* genes in sixteen NGS transcriptomic datasets of core *eduicots*. Six of them were from *Asterids*: *H. japonica*, *Panax genseng, Camellia sinensis, Schima mertensiana, Artemisia annua* and *Sesamum indicum*. Sequences of orthologs of these 13 genes in 14 transcriptomes were added to the alignment. The whole set was checked for frame shifts and if present, then corrected by inserting N to recover in frame translation. Translated protein sequences for the orthologs were made using EMBOSS [35] and aligned using MUSCLE [38]. The corresponding coding DNA sequence alignment was calculated using the program RevTrans1.4 [39]. Poorly aligned positions were removed using Gblocks (http://molevol.cmima.csic.es/castresana/Gblocks.html) with all options for a less stringent selection. Amino acid sequence alignments and nucleotide alignments were used to produce phylogenies using maximum parsimony (MP) and maximum likelihood (ML). The GTR + I + T model of nucleotide substitution were determined by the Akaike information criterion (AIC) in Modeltest ver. 3.7 [40]. The JTT model of amino acid substitutions was determined by the AIC in ModelGenerator for ML analysis. The Maximum Likelihood analysis was explored using PhyML 3.0 [41] for both to the amino acid and nucleotide data. The non-parametric bootstrap analysis was performed with 1000 replicates. The phylogenetic analysis using the Maximum Parsimony (MP) method was performed using PAUP* ver. 4. 0b8 [42]. The MP analysis involved a heuristic search using TBR branch swapping and 20 random addition replicates. Support for nodes was also evaluated with 1000 replicates of non-parametric bootstrapping.

## Competing interests

The authors declare that they have no competing interests.

## Authors’ contributions

CS and GY performed the experiments. CS drafted the manuscript. BZ, MB and GN finalized the paper. GN supervised the project. All authors read and approved the final manuscript.

## Supplementary Material

Additional file 1 **All of the assembled unigenes derived from *H. Willd.***
Click here for file

Additional file 2 **The mapped metabolic pathways of part of unigenes derived from *H. Willd.***
Click here for file

Additional file 3 The shared 13 sing-copy nuclear genes sequence from 83-taxon species. Click here for file

Additional file 4 Alignment of 13 single copy genes sequence in 83-taxon species. Click here for file
